# Respiratory syncytial virus infection, non‐respiratory syncytial virus respiratory infections, and later wheezing

**DOI:** 10.1111/ped.70050

**Published:** 2025-05-26

**Authors:** Ippei Takahashi, Genki Shinoda, Fumihiko Ueno, Fumiko Matsuzaki, Aoi Noda, Keiko Murakami, Mami Ishikuro, Taku Obara, Yoshikazu Nakayama, Atsushi Momose, Naho Tsuchiya, Satoshi Nagaie, Soichi Ogishima, Gen Tamiya, Nobuo Fuse, Atsushi Hozawa, Junichi Sugawara, Shigeo Kure, Shinichi Kuriyama

**Affiliations:** ^1^ Division of Molecular Epidemiology, Graduate School of Medicine Tohoku University Sendai Japan; ^2^ Department of Preventive Medicine and Epidemiology Tohoku Medical Megabank Organization, Tohoku University Sendai Japan; ^3^ Department of Pharmaceutical Sciences Tohoku University Hospital Sendai Japan; ^4^ R&D Clinical Science Div Janssen Pharmaceutical K.K. Tokyo Japan; ^5^ Department of Health Record Informatics, Tohoku Medical Megabank Organization Tohoku University Sendai Japan; ^6^ Department of Integrative Genomics, Tohoku Medical Megabank Organization Tohoku University Sendai Japan; ^7^ Center for Advanced Intelligence Project RIKEN Tokyo Japan; ^8^ Division of Epidemiology, Graduate School of Medicine Tohoku University Sendai Japan; ^9^ Division of Personalized Prevention and Epidemiology, Graduate School of Medicine Tohoku University Sendai Japan; ^10^ Suzuki Memorial Hospital Iwanuma Japan; ^11^ Miyagi Children's Hospital Sendai Japan; ^12^ International Research Institute of Disaster Science Tohoku University Sendai Japan

**Keywords:** birth cohort, epidemiology, respiratory syncytial virus, RSV, wheezing

## Abstract

**Background:**

Studies investigating whether respiratory syncytial virus (RSV) infection, non‐RSV respiratory infections, respiratory‐related disorders, and non‐respiratory‐related disorders are associated with subsequent wheezing are limited in Japan. We aimed to elucidate the relationship between hospitalization for RSV infection, non‐RSV respiratory infections, respiratory‐related disorders, as well as non‐respiratory‐related disorders and subsequence wheezing in Japanese children.

**Methods:**

This study included 7340 children and was conducted under the TMM BirThree Cohort Study (Tohoku Medical Megabank Project Birth and Three‐Generation Cohort Study). Data was collected from birth records and questionnaires. We categorized hospitalization history into five categories: “no hospitalization,” hospitalizations for “RSV infection,” “non‐RSV respiratory infections,” “respiratory‐related disorders,” and “non‐respiratory‐related disorders.” The association of the five categories with later wheezing at 3 years of age was evaluated using multivariable logistic regression analysis.

**Results:**

After adjusting for covariates, an association was shown between hospitalization under 2 years of age and later wheezing (odds ratio [OR] = 2.78; 95% confidence interval [CI] = 1.97–3.88 for “RSV infection”; OR = 2.61; 95% CI = 1.44–4.57 for “non‐RSV respiratory infections”; and OR = 3.33; 95% CI = 2.43–4.54 for “respiratory‐related disorders”).

**Conclusion:**

Hospitalization of children under 2 years of age for RSV infection as well as non‐RSV respiratory infections and respiratory‐related disorders were associated with subsequent wheezing.

## INTRODUCTION

The respiratory syncytial virus (RSV) is one of the most commonly identified viruses in infants and young children as a cause of acute lower respiratory infection.^1^, ^2^, ^3^ More than half of children are reported to have been infected with RSV by 1 year of age, and nearly 100% by 2 years of age.^1^ Symptoms are usually mild, but infants and young children are potentially susceptible to more serious symptoms such as RSV‐associated lower respiratory tract infection (RSV‐LRTI).^3^, ^4^ Children with LRTI require hospitalization and management because of the decrease in blood oxygen levels. In addition, several previous studies reported that infants or young children with severe RSV have a higher risk of wheezing and asthma later in life.^5^, ^6^, ^7^, ^8^, ^9^, ^10^, ^11^, ^12^, ^13^, ^14^, ^15^, ^16^, ^17^

In Japan, RSVs are defined as Class V infections and are monitored under the National Epidemiology Surveillance of Infectious Diseases (NESID).^18^ In addition, RSV are reported by the Pediatric Sentinel Surveillance System; the NESID program provides the Infectious Diseases Weekly Report (IDWR) and the Infectious Agents Surveillance Report (IASR) by the National Institute of Infectious Diseases (NIID).^18^ Although the existence of nationwide RSV surveillance,^18^ the association between RSV and the risk of subsequent wheezing have been studied globally.^5^, ^6^, ^7^, ^8^, ^9^, ^10^, ^11^, ^12^, ^13^, ^14^, ^15^, ^16^, ^17^ To the best of our knowledge, published studies on this topic in Japan were limited.^15^, ^16^, ^17^ Therefore, a replication study in a different Japanese population would be necessary to build evidence in Japan.

Clarifying whether the association of LRTI, such as with RSV infections with wheezing, is characteristic of RSV‐LRTI only, or whether similar associations are seen with non‐RSV respiratory infection or others, may help to understand the etiology. While there are several studies examined not only RSV‐LRTI but also others,^13^, ^14^, ^17^ as far as we know, studies in Japan were limited.^17^ In light of the above, studies are needed that include both RSV and other respiratory infections and verify the previous findings on a larger scale in the population of Japan.

Given these circumstances, we investigated and considered the association of RSV infection, non‐RSV respiratory infections, respiratory‐related disorders, and non‐respiratory‐related disorders with later wheezing in the Tohoku Medical Megabank Project Birth and Three‐Generation Cohort Study (TMM BirThree Cohort Study).

## METHODS

### Study design and population

**FIGURE 1:**
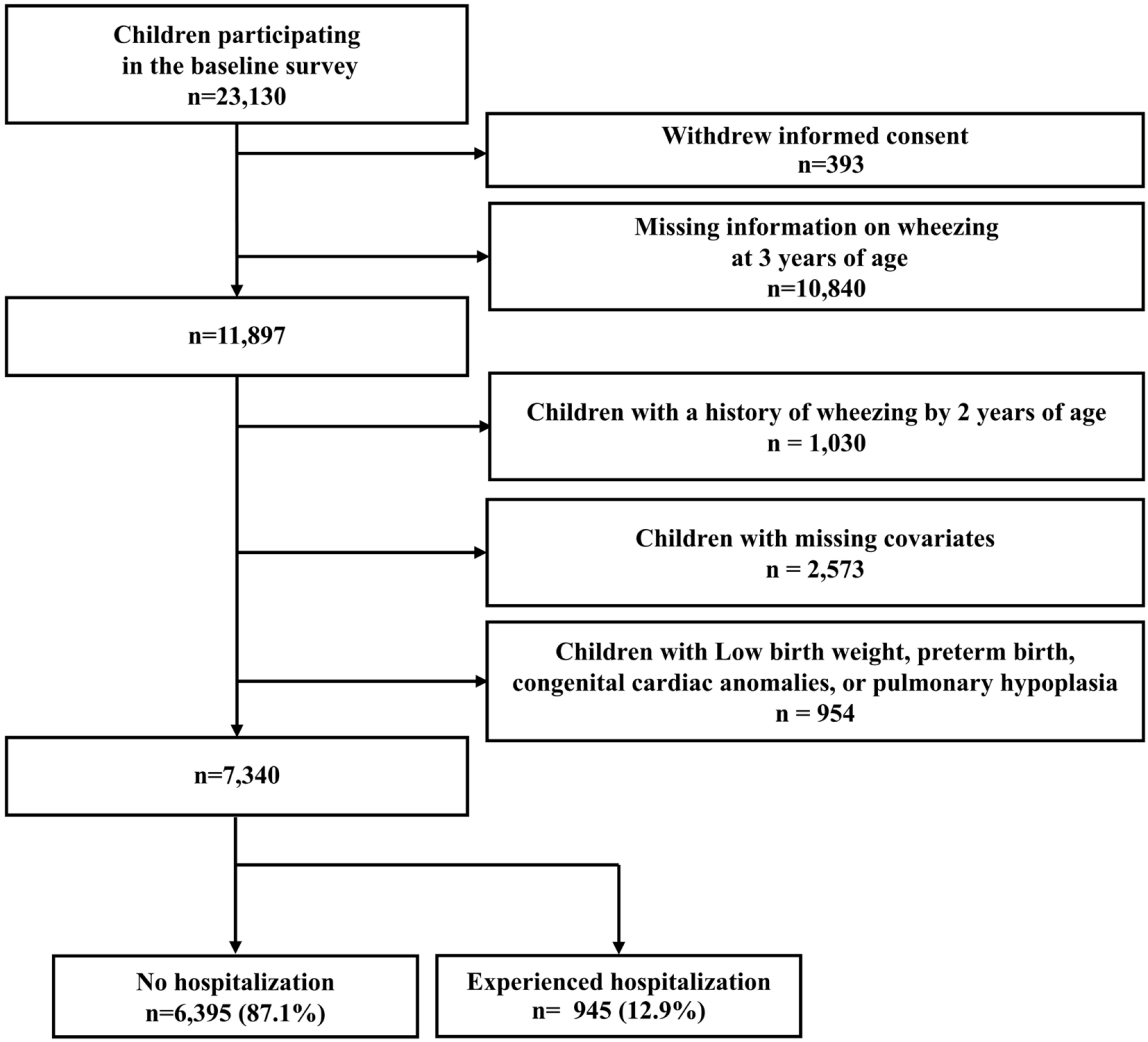
Flowchart of exclusion criteria for participants in this study.

The present study was conducted under the TMM BirThree Cohort Study. The TMM BirThree Cohort Study has been previously described.^19^, ^20^, ^21^, ^22^ Briefly, pregnant women that visited about 50 obstetric clinics and hospitals in Miyagi and Iwate prefectures, Japan were recruited into the TMM BirThree Cohort Study between July 2013 and March 2017.^19^, ^20^ Trained genomic medical research coordinators explained the details of the TMM BirThree Cohort Study to all potential participants and signed consent was obtained.^19^, ^20^ The protocol was reviewed and approved by the Institutional Review Board of the Tohoku Medical Megabank Organization (May 27, 2013; Approval No: 2013‐1‐103‐1). In the present study, the exclusion criteria applied to 23,130 children were as follows (Figure 1): withdrawn informed consent (*n* = 393), missing information on wheezing at 3 years of age (*n* = 10,840), children with a history of wheezing by the age of 2 (*n* = 1030), children with missing covariates (*n* = 1965). In addition, children with low birth weight, preterm birth, congenital cardiac anomalies, or pulmonary hypoplasia to focus on the onset of wheezing in children without risk factors for severe RSV infection (*n* = 954) were excluded. Of the 7340 children, 6395 children were never hospitalized under 2 years of age and 945 children were hospitalized under 2 years of age (Figure 1).

### Hospitalization of children

The history of hospitalization was determined using the health and lifestyle questionnaire responded by children's parents when the children were 1 month, 6 months, 1 year, and 2 years old. If a child's parent indicated that their child had been hospitalized within the past month, 6 months, or a year, the parents responded to a questionnaire on the history of hospitalization, which was used to determine the reason for hospitalization; the parents answered the corresponding categories regarding the name of the illness, the medical institution where the child was admitted, and the date of admission. We have categorized hospitalization history into five categories: no hospitalization, hospitalization for RSV infection, non‐RSV respiratory infections (respiratory infections not caused by RSV in which the causative agent has been identified), respiratory‐related disorders (chronic respiratory diseases such as asthma, airway narrowing, bronchitis, and pneumonia as well as diseases caused by bacterial or viral infections but for which the causative pathogen has not been identified), and non‐respiratory‐related disorders. There were no participants who were categorized in more than one category.

### Wheezing

The wheezing of children 2–3 years old was assessed using the International Study of Asthma and Allergies in Childhood (ISAAC) questionnaire.^23^ Children who were presented with wheezing before the age 2 were excluded because we aimed to evaluate new‐onset wheezing after the age of 3 years. This exclusion criterion was based on the premise that almost all infants and young children are infected with RSV by the age of 2 years.

### Covariates

Previously reported covariates^5^, ^6^, ^7^, ^8^, ^9^, ^10^, ^11^, ^12^, ^13^, ^14^, ^15^, ^16^, ^17^ that may influence the association between hospitalization of children under 2 years of age with subsequent wheezing at 3 years of age were selected: children's sex, living with older siblings at the age of 2 years, living with younger siblings at the age of 2 years, use of childcare facilities at the age of 2 years, passive smoking at the age of 2 years, maternal history of asthma, allergic rhinitis, and atopic dermatitis.

### Statistical analysis

The characteristics of the children were described according to five categories of hospitalization. The characteristics were presented as frequencies and percentages or mean and standard deviation. The association of the categories with subsequent wheezing at 3 years of age was evaluated using multivariable logistic regression analysis, and odds ratios (ORs) and 95% confidence intervals (CIs) were estimated using the “no hospitalization” category as the reference. All statistical analyses were performed using R version 4.0.2, and 95% CIs not crossing 1.00 were considered statistically significant.

## RESULTS

### Characteristics of the study population

The participant (*n* = 7340) characteristics are shown in Table 1. The number of children within each category was as follows: 6395 (87.1%) in “no hospitalization,” 176 (2.4%) in hospitalization with “RSV infection,” 58 (0.8%) in hospitalization with “non‐RSV respiratory infections,” 190 (2.6%) in hospitalization with “respiratory‐related disorders,” and 521 (7.1%) in “non‐respiratory‐related disorders.” In addition, 1051 (14.3%) children experienced wheezing at 3 years of age (Table 1).

**TABLE 1:** Characteristics of participants according to hospitalization status before 2 years of age.

	Total *n* = 7340	Hospitalization up to two years of age				
		No hospitalization *n* = 6395	RSV infection *n* = 176	Non‐RSV respiratory infections *n* = 58	Respiratory‐related disorders *n* = 190	Non‐respiratory‐related disorders *n* = 521
Sex, *n* (%)						
Male	3729 (50.8)	3202 (50.1)	91 (51.7)	39 (67.2)	107 (56.3)	290 (55.7)
Living with older siblings, *n* (%)						
Yes	3.895 (53.1)	3356 (52.5)	128 (72.7)	36 (62.1)	123 (64.7)	252 (48.4)
Living with younger siblings, *n* (%)						
Yes	719 (9.8)	633 (9.9)	14 (8.0)	9 (15.5)	17 (8.9)	46 (8.8)
Use of childcare facilities, *n* (%)						
Yes	3416 (46.5)	2906 (45.4)	95 (54.0)	34 (58.6)	121 (63.7)	260 (49.9)
Passive smoking at age 2, *n* (%)						
Yes	337 (4.6)	296 (4.6)	9 (5.1)	0 (0.0)	10 (5.3)	22 (4.2)
Maternal history of asthma, *n* (%)						
Yes	554 (7.5)	462 (7.2)	11 (6.2)	7 (12.1)	21 (11.1)	53 (10.2)
Maternal history of allergic rhinitis, *n* (%)						
Yes	1.800 (24.5)	1545 (24.2)	47 (26.7)	13 (22.4)	60 (31.6)	135 (25.9)
Maternal history of atopic dermatitis, *n* (%)						
Yes	1011 (13.8)	866 (13.5)	28 (15.9)	10 (17.2)	33 (17.4)	74 (14.2)
Wheezing, *n* (%)						
Yes	1051 (14.3)	829 (13.0)	52 (29.5)	18 (31.0)	69 (36.3)	83 (15.9)

*Note*: Statistics: *n* (%) and mean (standard deviation).

### Multivariable logistic regression analysis for hospitalization of children under 2 years of age and subsequent wheezing at 3 years of age

Table 2 shows the association between each category of hospitalization and subsequent wheezing at 3 years of age. After adjusting for covariates, an association between hospitalization under 2 years of age for RSV infection, non‐RSV respiratory infections, and respiratory‐related disorders (OR = 2.78, 95% CI: 1.973.88; OR = 2.61, CI: 1.44–4.57, and OR = 3.33, CI: 2.43–4.54, respectively) and risk of wheezing at 3 years of age. In contrast, non‐respiratory‐related disorders were not associated with subsequent wheezing at 3 years of age (OR = 1.18; 95% CI: 0.92–1.51).

**TABLE 2:** Association between five categories of hospitalizations under 2 years of age and subsequent wheezing at 3 years of age.

	Crude OR 95% CI	Adjusted OR^a^ 95% CI
*No hospitalization*	Reference	Reference
*Hospitalization by*		
RSV infection	**2.82 (2.01, 3.90)**	**2.78 (1.97, 3.88)**
Non‐RSV respiratory infections	**3.02 (1.68, 5.21)**	**2.61 (1.44, 4.57)**
Respiratory‐related disorders	**3.83 (2.81, 5.18)**	**3.33 (2.43, 4.54)**
Non‐respiratory‐related disorders	1.27 (0.99, 1.62)	1.18 (0.92, 1.51)

Abbreviations: CI, confidence interval; OR, odds ratio; RSV, respiratory syncytial virus.

*Note:* Categories in which an association has been detected are shown in bold.

^a^ Adjusted for sex, living with older siblings at age 2, living with younger siblings at age 2, use of childcare facilities at age 2, passive smoking at two years of age, maternal history of asthma, allergic rhinitis, and atopic dermatitis.

## DISCUSSION

In this study, hospitalization for “RSV infection,” “non‐RSV respiratory infections,” and “respiratory‐related disorders” under 2 years of age were significantly associated with subsequent wheezing at 3 years of age. These results emphasize not only hospitalizations for RSV respiratory infection but also hospitalizations for non‐RSV respiratory infections and respiratory‐related disorders associated with subsequent wheezing. On the other hand, the prevalence of hospitalization due to RSV infection is as high as 2.4%, suggesting that RSVs account for a burden on healthcare resources.

The present findings support previous studies that have shown an association of LRTI, such as with RSV infections with subsequent wheezing.^5^, ^6^, ^7^, ^8^, ^9^, ^10^, ^11^, ^12^, ^13^, ^14^, ^15^, ^16^, ^17^ In this study, of the 7340 participants in the analysis, 176 (2.4%) were hospitalized for RSV infection. A study using the Japan Medical Data Center (JMDC) database estimated that 3 to 4 out of every 100 Japanese children younger than 6 months are hospitalized for RSV respiratory.^15^ Considering that children with wheezing by age 2 years were excluded from the analysis in the present study, there is probably not a large departure between the prevalence in the present study and that of the JMDC database. Therefore, it is highly possible that the present study was able to analyze a representative population of Japanese children.

Regarding the confirmed association in the categories of “non‐RSV respiratory infections” and “respiratory‐related disorders”, these results replicate a previous finding that hospitalization for respiratory infections other than RSV‐LRTI is also associated with subsequent wheezing.^13^, ^14^, ^17^ According to the above, hospitalizations for respiratory infections and respiratory disorders may have a common etiology for subsequent wheezing. One hypothesis that explains the association of respiratory infections and respiratory disorders with wheezing is respiratory infection‐induced bronchial hyperresponsiveness.^9^, ^24^, ^25^ Therefore, the results of the present study might be explained by increased bronchial hyperresponsiveness as a possible etiological factor. In particular, the fact that no association was found in the “non‐respiratory‐related hospitalization” category in this study may strengthen the hypothesis. Another hypothesis is that RSV‐LRTI is a clinical phenotype.^24^, ^26^ In other words, children who congenitally have a sensitive respiratory system and are susceptible to wheezing have developed respiratory infections from infancy to childhood.^24^, ^26^ In the future, causal modeling strategies that also use information on genetic susceptibility may be able to elucidate these inconclusive discussions.^26^

This study has two strengths: first, it used data from a prospective cohort study in Japan that had enough sample size to assess associations; second, it was able to distinguish not only hospitalizations due to RSV but also non‐RSV respiratory infections, respiratory‐related disorders, and non‐respiratory‐related disorders. This allowed us to examine whether subsequent wheezing was specifically associated with hospitalization due to RSV or not. A limitation of this study is that information on hospitalizations for “RSV infection,” “non‐RSV respiratory infections,” “respiratory‐related disorders,” and “non‐respiratory‐related disorders” under 2 years of age and wheezing at 3 years of age was collected by self‐report questionnaire, not by physician diagnosis. Therefore, misclassification cannot be completely excluded. In addition, assessing wheezing within the past 12 months at 3 years of age might capture a high incidence of wheezing due to respiratory infections other than asthma, resulting in an overall high prevalence of wheezing in the study population. Further follow‐up of children at older ages, when wheezing not associated with asthma is less common, may be able to overcome this limitation.

In conclusion, not only hospitalization for RSV infection but also hospitalization for non‐RSV respiratory infections and respiratory‐related disorders in children under 2 years of age were associated with subsequent wheezing at 3 years of age in Japan.

## AUTHOR CONTRIBUTIONS

I.P., T.O., and F.U. conceived and designed the study; all authors acquired, analyzed, or interpreted data; I.T. drafted the manuscript; all authors performed critical revision of the manuscript for important intellectual content; I.T. and F.U. performed statistical analysis; S. K. obtained funding; I.T., T.O., and S.K. provided administrative, technical, or material support; and S.K. supervised the study. All authors read and approved the final manuscript.

## CONFLICT OF INTEREST STATEMENT

This study was conducted in collaboration with Janssen Pharmaceutical K.K. The other authors declare no conflict of interest.

## Data Availability

The data that support the findings of this study are available from the TMM biobank; however, restrictions apply to the availability of these data, which were used under license for the current study and hence are not publicly available. Data are available from the authors upon reasonable request and with the permission of the TMM biobank. All inquiries about access to data should be sent to the TMM biobank (dist@megabank.tohoku.ac.jp).
